# A Combined Comprehensive Palliative and Rehabilitative Care Plan for a Child With Cerebral Palsy

**DOI:** 10.7759/cureus.75010

**Published:** 2024-12-03

**Authors:** Raktim Swarnakar, Shiv L Yadav

**Affiliations:** 1 Physical Medicine and Rehabilitation, R. G. Kar Medical College and Hospital, Kolkata, IND; 2 Physical Medicine and Rehabilitation, All India Institute of Medical Sciences, New Delhi, New Delhi, IND

**Keywords:** cerebral palsy (cp), palliative care services, physical medicine and rehabilitation, physical medicine and rehabilitation (pm&r), rehabilitation program

## Abstract

Cerebral palsy (CP) is a group of neurological disorders that affect movement, muscle tone, and motor skills. Here, we present a case of an 11-year-old female patient who presented with tightness in both lower limbs, since birth, and delayed walking, accompanied by difficulty walking due to spasticity. She was diagnosed with spastic diplegic cerebral palsy. This case highlights the combined use of palliative and rehabilitative care. Developmental milestones were delayed, and she was diagnosed with CP at an early age. A comprehensive treatment plan was implemented, including physical therapy, speech therapy, and pharmacological treatment with baclofen to reduce spasticity. Non-pharmacological treatments such as neurodevelopmental therapy, biofeedback, and orthotic support were also employed. The child’s rehabilitation progress included improved mobility, speech fluency, and reduced spasticity, with notable benefits in gait. Palliative care played a critical role in addressing the emotional, psychological, and social aspects of the child's condition and providing the family support, counseling, and guidance on managing the care burden. This integrated approach emphasizes the importance of early palliative care alongside rehabilitation to optimize a child’s quality of life. This case highlights how a multidisciplinary approach addressing both physical and emotional needs can lead to better overall outcomes for children with cerebral palsy, improving not just mobility but also their well-being and family support system.

## Introduction

Cerebral palsy (CP) is a group of neurological disorders that affect movement, muscle tone, and motor skills, with a global prevalence estimated at two to three cases per 1,000 live births [1,2]. As CP has an impact on a child’s physical, emotional, and social well-being, it requires a multidisciplinary approach to care [3]. Traditionally, CP treatment has focused on alleviating physical symptoms through medical interventions. However, given the variability in the complexity of CP from case to case, there has been increasing recognition of the importance of combining palliative and rehabilitative care [4,5]. This integrated approach aims not only to manage the physical challenges faced by children with CP but also to enhance their quality of life, support their families, and address their psychological and emotional needs [3]. This case report explores the application of a comprehensive framework combining palliative and rehabilitative care for a child with cerebral palsy, highlighting the benefits and challenges of this multidisciplinary strategy. The case further emphasizes the significance of early intervention, personalized care plans, and a holistic approach to managing the diverse aspects of living with CP.

## Case presentation

The patient was an 11-year-old girl with a history of tightness in both lower limbs since birth and delayed walking, starting at three years of age. The parents observed difficulty with walking, particularly using both her legs due to tightness, although the issue was non-progressive and without falls or weakness. Born prematurely at 32 weeks of gestation with a delayed cry, she had experienced developmental delays, including delayed motor milestones such as sitting at 12 months and walking at three years. The child had normal bowel, bladder, sleep, and appetite functions, and her family history was unremarkable for similar illnesses.

In terms of functionality, she walked with limitations (Gross Motor Function Classification System level II), handled objects at a reduced speed (Manual Ability Classification System level II), and communicated with extra time needed. She began school at the age of 7, and showed an interest in drawing. A physical examination revealed an average nutritional status with no signs of anemia, icterus, or other systemic issues. Her growth parameters were within normal ranges, with a weight of 27 kg and height of 138 cm. The diagnosis was spastic diplegic cerebral palsy with dysarthria.

**Figure 1:**
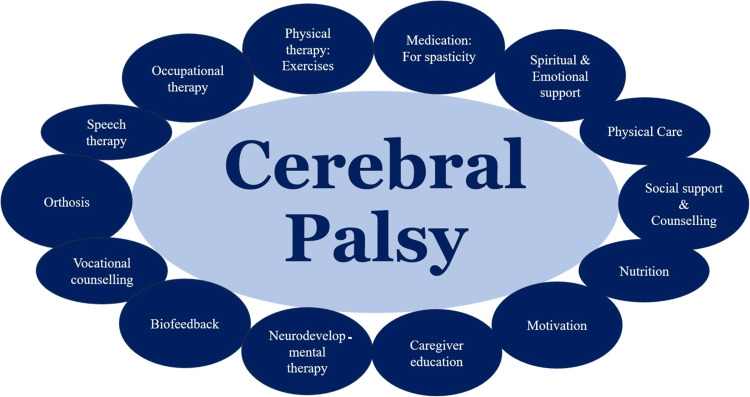
Comprehensive palliative and rehabilitative care plan for cerebral palsy

She received a comprehensive treatment plan that included both palliative and rehabilitative care, combining medical and therapeutic interventions (Figure 1) [4,6].

The patient and her parents were educated about the condition, and goal-setting was done to help address physical, social, and spiritual care needs. Counseling sessions were held to help the family understand the condition and cope with the challenges of cerebral palsy, while emotional support was provided to address any stress or frustration related to the child’s developmental delays and physical limitations. Additionally, strategies were discussed to manage daily challenges and foster a positive outlook, along with education provided on caregiver burden and the importance of maintaining their own health.

Baclofen (15 mg/day) was prescribed to reduce spasticity, and various exercises, including stretching and strengthening exercises for the limbs and spinal muscles, were implemented. Speech therapy, biofeedback for gait training, and neurodevelopmental therapy (Bobath approach) were also included as part of the non-pharmacological treatment plan [5,6]. Bilateral ankle foot orthoses (AFOs) were provided to support walking, and home modifications were suggested to prevent falls. Vocational counseling was offered to address school-related concerns. Additional evaluations by ENT, ophthalmology, physiotherapy, occupational therapy, and speech therapy were conducted to further support her development and treatment.

The patient has made significant progress in several areas since her first presentation. She is now walking independently with the help of bilateral AFOs as shoe inserts. There has been a noticeable improvement in speech fluency; spasticity has been reduced, and it no longer impedes activities of daily living, though it has not fully disappeared. Biofeedback has also contributed to a considerable improvement in her gait, which is much better than before.

## Discussion

Palliative care should begin at the diagnosis of cerebral palsy, as it addresses not only the physical but also the psychosocial, emotional, and spiritual needs of the patient [4]. Research suggests that many children with CP in India require pediatric palliative care. Alongside pediatric rehabilitation, care should focus on improving communication with the child and family, providing counseling, addressing developmental issues, and educating about home care. This holistic approach encompasses physical, psychosocial, and spiritual care, while also supporting caregivers emotionally and financially [7,8]. At both personal and professional levels, it is clear that comprehensive care goes beyond medications and exercise, integrating the child’s mind, body, and spirit. Effective communication and the early initiation of palliative care, alongside rehabilitation, are essential to improving outcomes. Furthermore, addressing ethical concerns, such as respecting autonomy and avoiding harm, ensures the best possible care for the patient.

In this case, the integration of palliative and rehabilitative care was crucial for optimizing the child’s overall well-being [3]. CP affects multiple aspects of a child’s life, and while rehabilitation primarily focuses on improving motor function, palliative care addresses a broader spectrum of needs, including physical, emotional, and social aspects. Though rehabilitative therapies like speech therapy, physiotherapy, and orthotic support aim to improve function and mobility, they do not fully address the emotional burden or challenges associated with developmental delays and spasticity. Palliative care plays an essential role in alleviating discomfort, managing chronic pain, and enhancing quality of life, complementing the rehabilitation process.

While rehabilitation improves physical abilities such as walking and communication, palliative care ensures that the child’s comfort is prioritized, particularly in terms of reducing spasticity, managing pain, and preventing secondary complications. In this case, baclofen was prescribed to reduce spasticity, and regular monitoring was recommended for emerging issues like chronic pain or infections. These measures are vital in managing a non-progressive yet life-limiting condition like cerebral palsy, where the goal is not just improving mobility but also ensuring that the child is as comfortable as possible in their day-to-day life.

The integration of both care models offers a holistic approach. While rehabilitation enhances motor skills, palliative care addresses the mental, emotional, and social consequences of the condition. The child’s struggle with developmental delays and gait abnormalities likely affected her self-esteem and social integration. Palliative care provides counseling and emotional support to both the child and family, helping them navigate the psychological challenges of living with cerebral palsy. Family involvement is a core component of palliative care, as caregivers often face significant emotional and financial strain [9,10]. Offering emotional support and practical guidance on home care allows the family to better manage their role as caregivers while also addressing their own needs, reducing stress, and creating a positive environment for the child.

Ethically, the dual approach of palliative and rehabilitative care ensures that treatment is not solely focused on improving motor function but also considers the child’s overall comfort and well-being. Palliative care ensures that interventions, such as baclofen use, are carried out with the child’s quality of life in mind, carefully weighing the benefits and potential risks. It also respects the autonomy of the child and family, ensuring they are actively involved in decisions about care, treatment plans, and future goals.

Addressing the emotional and psychological needs of the child and family is another critical aspect of palliative care. The child’s developmental delay and difficulty in understanding her condition could lead to feelings of frustration or anxiety. Palliative care helps manage these feelings, offering coping strategies and emotional support to both the child and family members. In this case, while rehabilitation improves physical function, palliative care ensures that the child’s emotional and social needs are also met, providing a balanced and compassionate approach to care.

A combined approach of palliative and rehabilitative care is essential in managing the complexities of cerebral palsy. While rehabilitation improves physical abilities and functional outcomes, palliative care addresses the comprehensive needs of the child and their family, including emotional, social, and psychological support. This holistic care model ensures that the child not only benefits from enhanced mobility and function but also experiences improved comfort, emotional well-being, and quality of life. Integrating both care models offers the best possible support for children with cerebral palsy, ensuring that all aspects of their health are addressed in a compassionate and effective manner [11].

## Conclusions

This case shows the significance of integrating both palliative and rehabilitative care in the management of children with cerebral palsy. While rehabilitative interventions greatly enhance physical function and mobility, palliative care focuses on the child’s overall well-being by addressing emotional, psychosocial, and comfort-related concerns. A holistic, multidisciplinary approach that includes pharmacological treatments, physical therapy, speech therapy, family support, and psychological counseling significantly improves the quality of life for both the child and their family. Early initiation of palliative care, alongside rehabilitation, is crucial for maximizing comfort, minimizing distress, and promoting independence. Ultimately, this approach fosters a more comprehensive and compassionate care plan for children with complex, life-long conditions such as cerebral palsy.
